# Machine Learning in Identifying Marker Genes for Congenital Heart Diseases of Different Cardiac Cell Types

**DOI:** 10.3390/life14081032

**Published:** 2024-08-19

**Authors:** Qinglan Ma, Yu-Hang Zhang, Wei Guo, Kaiyan Feng, Tao Huang, Yu-Dong Cai

**Affiliations:** 1 School of Life Sciences, Shanghai University, Shanghai 200444, China; mql1117@shu.edu.cn; 2 Channing Division of Network Medicine, Brigham and Women’s Hospital, Harvard Medical School, Boston, MA 02115, USA; reyhz@channing.harvard.edu; 3 Key Laboratory of Stem Cell Biology, Shanghai Jiao Tong University School of Medicine (SJTUSM) & Shanghai Institutes for Biological Sciences (SIBS), Chinese Academy of Sciences (CAS), Shanghai 200030, China; gw_1992@sjtu.edu.cn; 4 Department of Computer Science, Guangdong AIB Polytechnic College, Guangzhou 510507, China; kyfeng@gdaib.edu.cn; 5 Bio-Med Big Data Center, CAS Key Laboratory of Computational Biology, Shanghai Institute of Nutrition and Health, University of Chinese Academy of Sciences, Chinese Academy of Sciences, Shanghai 200031, China; 6 CAS Key Laboratory of Tissue Microenvironment and Tumor, Shanghai Institute of Nutrition and Health, University of Chinese Academy of Sciences, Chinese Academy of Sciences, Shanghai 200031, China

**Keywords:** congenital heart disease, single cell, cardiac cell, machine learning, marker gene

## Abstract

Congenital heart disease (CHD) represents a spectrum of inborn heart defects influenced by genetic and environmental factors. This study advances the field by analyzing gene expression profiles in 21,034 cardiac fibroblasts, 73,296 cardiomyocytes, and 35,673 endothelial cells, utilizing single-cell level analysis and machine learning techniques. Six CHD conditions: dilated cardiomyopathy (DCM), donor hearts (used as healthy controls), hypertrophic cardiomyopathy (HCM), heart failure with hypoplastic left heart syndrome (HF_HLHS), Neonatal Hypoplastic Left Heart Syndrome (Neo_HLHS), and Tetralogy of Fallot (TOF), were investigated for each cardiac cell type. Each cell sample was represented by 29,266 gene features. These features were first analyzed by six feature-ranking algorithms, resulting in several feature lists. Then, these lists were fed into incremental feature selection, containing two classification algorithms, to extract essential gene features and classification rules and build efficient classifiers. The identified essential genes can be potential CHD markers in different cardiac cell types. For instance, the LASSO identified key genes specific to various heart cell types in CHD subtypes. *FOXO3* was found to be up-regulated in cardiac fibroblasts for both Dilated and hypertrophic cardiomyopathy. In cardiomyocytes, distinct genes such as *TMTC1*, *ART3*, *ARHGAP24*, *SHROOM3*, and *XIST* were linked to dilated cardiomyopathy, Neo-Hypoplastic Left Heart Syndrome, hypertrophic cardiomyopathy, HF-Hypoplastic Left Heart Syndrome, and Tetralogy of Fallot, respectively. Endothelial cell analysis further revealed *COL25A1*, *NFIB*, and *KLF7* as significant genes for dilated cardiomyopathy, hypertrophic cardiomyopathy, and Tetralogy of Fallot. LightGBM, Catboost, MCFS, RF, and XGBoost further delineated key genes for specific CHD subtypes, demonstrating the efficacy of machine learning in identifying CHD-specific genes. Additionally, this study developed quantitative rules for representing the gene expression patterns related to CHDs. This research underscores the potential of machine learning in unraveling the molecular complexities of CHD and establishes a foundation for future mechanism-based studies.

## 1. Introduction

Congenital heart disease (CHD) encompasses a variety of inborn heart defects that affect the structure and function of the heart [1,2]. Medically, the entity of CHD can be accurately described as a pathological condition initiated by the dysfunction of heart arteries, causing insufficient oxygen supply through blood to the heart. CHD can significantly impact the circulatory system, leading to altered blood flow throughout the patient’s life [3]. The disease presents in several forms, each with distinct pathological characteristics and clinical manifestations. Some patients with CHD can have very mild symptoms. However, CHD can also be deadly and is shown to be the leading cause of death in infants, children, and adolescents [4,5]. In the United States, according to Circulation’s report in 2010 [5], about 1 million children and 1.4 million adults suffered from such disease ever since they were born. More than 10% of these people have severe CHD with missing or poorly formed heart parts [5], threatening their lives. 

Pathologically, CHD is a progressive heart condition with structural heart damage and/or blood vessel connection disorders involving heart wall impairment, heart valve dysfunction, and blood vessel flow abnormalities [6,7]. Medically, the causes of CHD can be summarized as atherosclerosis (plaque or atheroma on the walls of coronary arteries), vasospasm (inner wall damage of the coronary arteries, including spasm), and coronary microvascular disease (smaller blood vessel dysfunction). The defect of heart functions induces common symptoms of CHD, including cyanosis, fatigue, unusual breath, heart murmur, and poor blood circulation. Molecularly, human CHD is generally induced by two directions of abnormalities: (1) heart development: genes like *NKX2-5* and *GATA4* and pathways like Wnt, Notch, and BMP participate in human CHD pathogenesis by disrupting human heart development [8,9]; (2) heart structural abnormalities: fibrillin dysfunction leads to abnormal protein production, affecting the heart’s scaffolding and structural integrity [10], which further initiates CHD. The explanation of human CHD pathogenesis is a challenge due to its molecular complexity as follows: firstly, although for individuals, the initiation of CHD is generally induced by only one gene, the single-gene abnormality can induce different symptoms; secondly, the same symptoms, like heart valve dysfunction, can be caused by the combination of different genetic variations; thirdly, different subtypes of CHD share some similar symptoms and even molecular pathogenesis, making it hard to conduct CHD clustering.

Currently, clinical treatment of CHD can be summarized into three major aspects [1,11]: (1) Firstly, surgery, which is the most effective, but with the highest risk, highest cost, and severe physical trauma. The major approaches for CHD surgeries include open heart surgery (allows the surgeon to repair the heart), catheterization (expands narrowed heart arteries), and balloon valvuloplasty (a specific balloon-medicated catheterization). (2) Secondly, medications, which can only help regulate blood flow statuses like beta blockers, calcium channel blockers, angiotensin-converting enzyme inhibitors, nitroglycerin, ranolazine, antiplatelets, and antihyperlipidemics. For long-term monitoring and treatment, patients need to take the medicine constantly throughout their lives, which is not only time and cost-consuming but also causes damage to the liver and other organs. (3) Catheterizations, which are also expensive, time-consuming, and have only time-limited effects. Therefore, due to the lack of effective treatment for CHD, disease prevention is quite important. The only approach to this is to reduce heart disease risk and detect CHD at an early stage. Heart disease risk can be managed through blood pressure control, cholesterol control, and diabetes management, while the early detection of CHD requires regular checkups on heart health. However, there are no effective gene signatures for CHD interference reported. Therefore, finding potential gene signatures for CHD disease status monitoring and diagnosis is one of the most important scientific questions in the field of cardiovascular diseases.

CHD can be well-treated with definitive surgical correction to improve the survival rate [2]. However, CHD is a complex disease with different subtypes, including Tetralogy of Fallot (TOF) [12,13], hypoplastic left heart syndrome (HLHS, including neo-HLHS and HF-HLHS) [14], hypertrophic cardiomyopathy (HCM) [15], and dilated cardiomyopathy [16]. TOF is a complex condition involving four distinct defects: ventricular septal defect, pulmonary stenosis, right ventricular hypertrophy, and an overriding aorta, affecting the blood flow out of the heart [13]. HLHS is characterized by the underdevelopment of the left heart structures, leading to inadequate blood flow [14,17]. HCM involves the thickening of the heart muscle, particularly the left ventricle, which can obstruct blood flow and lead to arrhythmias [18]. DCM is marked by the dilation and weakened contraction of the heart chambers, primarily the left ventricle, resulting in heart failure and arrhythmias [19]. Different CHD subtypes have different clinical phenotypes and molecular pathological mechanisms, which further require different clinical treatment strategies. For instance, TOF typically has to be surgically corrected post-birth. The surgical repair of heart defects involves reconstruction of the heart to promote oxygenation and circulatory processes in affected children. It may dramatically raise both the survival and good living rates among such children [20]. DCM is treated using drugs like ACE inhibitors or beta-blockers to manage pressure levels and promote blood circulation while also lowering the strain on the heart. A heart transplant is recommended for extreme cases [21]. HLHS is treated by staged reconstructive surgeries, including the Norwood, Glenn, and Fontan procedures, which help to rebuild the heart sufficiently to pump blood [22,23]. HCM is treated primarily with drugs such as beta-blockers and calcium channel blockers; however, in severe cases, options such as septal myectomy surgery can improve blood circulation [24]. Therefore, revealing the different molecular mechanisms for different CHD subtypes can also help establish more effective medication guidelines for CHD treatment. Various single-cell transcriptomics analyses have been performed in CHD to reveal the molecular mechanisms of different CHD subtypes at the single-cell level. In 2022, Miranda and Noseda from Imperial College London reviewed recent updates on single-cell CHD analyses [25]. Various cell signatures have been recognized in cardiomyocytes, fibroblasts, vascular cells, immune cells, and even some rare cell types. In cardiomyocytes, genes like *B2M* in ischaemic injury [26], *EPHB1* in HCM [27], and *SH3RF2* in DCM [28] have been verified. In fibroblasts, *ACE2* and *ELN* are two key genes identified for CHD [29]. As for vascular cells, different key genes were found for endothelial cells and smooth muscle cells. *NRG3* and *BMP6* were found to be pathogenic in endothelial cells in DCM [30,31], while in muscle cells, *AGT* was found to be a potential biomarker in the same CHD subtype [32]. Immune cells include various cell types, which have different genes for CHD subtyping. In DCM hearts, *LINGO2* [33] was shown to be a lymphoid cell-specific gene. Therefore, CHD is a heterogeneous disease with different pathological phenotypes (clinical phenotypes) and single-cell-level expression profiling.

The heart is an important organ with various cell types, including cardiomyocytes, cardiac fibroblasts, endothelial cells, and mesothelial cells [34]. The cardiovascular defect phenotype of CHD has been shown to be associated with important heart cells, cardiomyocytes, cardiac fibroblast, and endothelial cells, which have also been further validated by in vitro recapitulation using patient iPSC-derived cardiac cells [35]. Different cell types may play different roles during pathogenesis, leading to the complexity of CHD. Among all the cells, cardiomyocytes [36], cardiac fibroblast [37], and endothelial cells [38] have been shown to be highly connected with CHD pathogenesis, according to previous publications. Therefore, in this study, based on the transcriptomics data from a recent multi-Omics study on CHD [14], we utilized multiple machine learning algorithms to extract key molecular factors for CHD subtyping using transcriptomics from three major cell types: cardiomyocytes (CM), cardiac fibroblast (CF), and endothelial cells (Endo). First, six feature-ranking algorithms, including categorical boosting (CatBoost) [39], least absolute shrinkage and selection operator (LASSO) [40], light gradient boosting machine (LightGBM) [41], Monte Carlo feature selection (MCFS) [42], random forest (RF) [43], and eXtreme gradient boosting (XGBoost) [44], were applied to the transcriptomics data on three cell types, yielding several feature lists. Then, these lists were fed into incremental feature selection (IFS) [45] one by one, which incorporated synthetic minority oversampling technique (SMOTE) [46] and two classification algorithms [decision tree (DT) [47] and RF] to extract essential features and classification rules and build optimal classifiers. Some identified gene features were analyzed. Compared with previous studies, we first tried to explain the complex mechanisms of CHD from the perspective of individual cell types. Our results can not only reveal the molecular differences between CHD subtypes but also recognize cell-type-specific contributions during CHD pathogenesis, providing a comprehensive transcriptomic regulatory atlas of the initiation and progression of CHD.

## 2. Materials and Methods

### 2.1. Data from the Single-Nucleus RNA Sequencing of Heart Tissues

**Figure 1:**
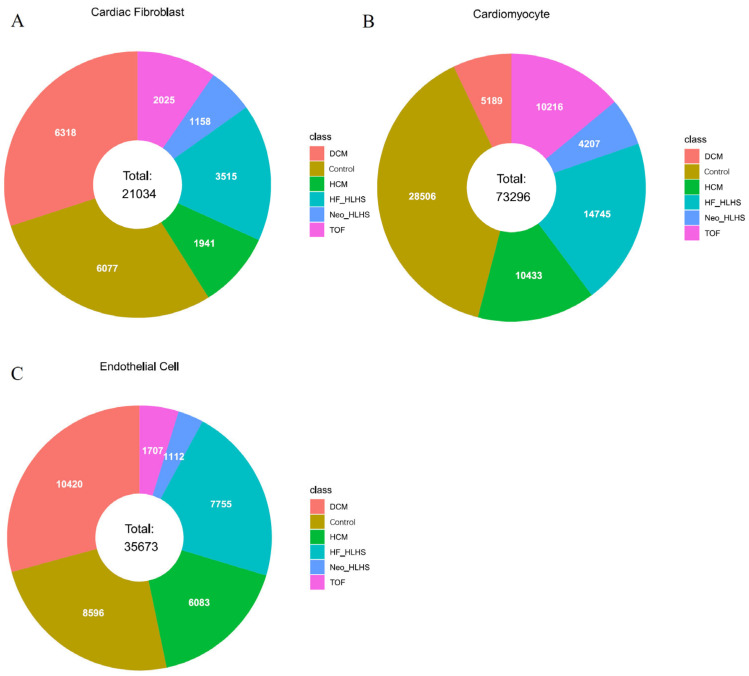
Pie chart of the data composition. This image details the number of samples for each of six CHD forms (DCM, control, HCM, HF_HLHS, Neo_HLHS, and TOF). It provides a quantitative overview essential for dataset analysis. (**A**) Data composition for cardiac fibroblast; (**B**) Data composition for cardiomyocytes; (**C**) Data composition for endothelial cells.

In this study, we utilized single-nucleus RNA sequencing data of cardiac cells sourced from Hill et al.’s study [14]. The dataset encompassed three cardiac cell types: CF, CM, and Endo, comprising 21,034, 73,296, and 35,673 cells, respectively. For CF, the dataset included cells from 6318 cases of dilated cardiomyopathy (DCM), 6077 donor hearts (used as healthy controls), 1941 cases of hypertrophic cardiomyopathy (HCM), 3515 cases of heart failure with hypoplastic left heart syndrome (HF_HLHS), 1158 neonatal cases of HLHS (Neo_HLHS), and 2025 cases of Tetralogy of Fallot (TOF). For CM, the counts were 5189 DCM, 28,506 controls, 10,433 HCM, 14,745 HF_HLHS, 4207 Neo_HLHS, and 10,216 TOF. Finally, for Endo, the samples comprised 10,420 DCM, 8596 controls, 6083 HCM, 7755 HF_HLHS, 1112 Neo_HLHS, and 1707 TOF cases. Figure 1 shows the composition of the data for three cardiac cell types. Each data piece contains 29,266 gene expressions as features.

### 2.2. Feature-Ranking Methods Used to Rank Features in Order of Importance

Our study delved into the gene expression profiles of specific samples, discovering numerous genes, but only a few linked to heart. To gain a deeper insight into these heart-associated genes, we employed six feature-ranking algorithms. These included CatBoost [39], LASSO [40], LightGBM [41], MCFS [42], RF [43], and XGBoost [44]. These methods have wide applications in tackling complicated data [48,49,50,51,52,53]. Each method provided a unique view to rank features, enhancing our understanding of the data and ensuring a thorough analysis of the significance of the identified genes.

#### 2.2.1. Categorical Boosting

In CatBoost [39], feature importance can be estimated by Prediction Values Change. It calculates the importance of a feature as the average change in the prediction value when this feature value changes. This is performed by shuffling the values of the feature and measuring the resulting decrease in the model’s performance. The more the performance decreases, the more important the feature is.

#### 2.2.2. Least Absolute Shrinkage and Selection Operator

Since LASSO [40] performs L1 regularization, it has the effect of shrinking some of the model’s coefficients toward zero, effectively “eliminating” them from the model. The variables with non-zero coefficients at the end of the LASSO procedure are typically interpreted as being important for the prediction task. Feature importance can be determined by the absolute value of the magnitude of the coefficients of the features.

#### 2.2.3. Light Gradient Boosting Machine

LightGBM is a gradient-boosting framework developed by Microsoft that uses tree-based learning algorithms [41]. There are two main types of feature importance provided by LightGBM: “split” and “gain”. (1) Split Importance: This represents the number of times a feature is used to split the data across all trees. (2) Gain importance: This measures the total gain of a feature when it is used in trees.

#### 2.2.4. Monte Carlo Feature Selection

MCFS generates a large array of decision trees, each of which is built by randomly selecting some features and data from the original dataset. The importance of a feature is then determined by the weighted accuracy, the information gain of each node split by the feature, and the coverage of each node [42]. 

#### 2.2.5. Random Forest

The traditional method for calculating feature importance in RF is Mean Decrease Impurity (MDI) [43]. For each tree in the forest, the decrease in the Gini impurity (or whichever metric is being used) that results from splitting on a particular feature is calculated and averaged over all trees. Higher MDI indicates that the feature is more important.

#### 2.2.6. eXtreme Gradient Boosting

XGBoost is an open-source library that provides a gradient-boosting framework [44] based on decision trees. XGBoost offers several methods to calculate feature importance, and they are summarized as follows: (1) Weight: This calculates the number of times a feature is used to split the data across all trees. (2) Gain: This is the average training loss reduction gained when using a feature for splitting. (3) Cover: This is the average coverage of instances affected by splits on this feature.

### 2.3. Incremental Feature Selection

Following the ranking of features using methods such as CatBoost, LASSO, LightGBM, MCFS, RF, and XGBoost, IFS is a feature-selection algorithm used to identify the optimal subset of features for a given machine learning model or task [45]. It is an iterative process where features are added one by one (or sometimes removed) in order and each time their performance is evaluated. Eventually, the feature subset that gives the best performance is chosen as the optimal feature subset. 

### 2.4. Synthetic Minority Oversampling Technique

It can be observed that three datasets were imbalanced. Some category was evidently larger than others. The classifiers built on such dataset may produce bias. SMOTE is a popular algorithm used to tackle this problem [46]. It is an oversampling method that creates synthetic (not duplicate) samples of the minority class. Essentially, it generates new samples by interpolating between samples of the minority class.

### 2.5. Classification Algorithm

In this study, we employed two supervised classification algorithms, DT and RF, to implement the IFS method. The DT algorithm [47] utilizes a hierarchical decision structure for classification. In contrast, RF [43] leverages multiple DTs to bolster accuracy. Both algorithms are instrumental in optimizing the IFS method by selectively refining critical features, thereby enhancing model performance.

### 2.6. Performance Evaluation

The weighted F1 score is a vital metric in machine learning, especially for handling class imbalances [54,55,56,57,58,59]. Unlike the macro F1 score, it considers class sizes, giving more weight to larger classes. This provides a detailed assessment of classifier performance in real-world scenarios, making it crucial for tasks like medical diagnosis and fraud detection, where imbalanced data is common and accurate evaluation is essential. The specific formula for this metric is as follows:

(1) $${Precision}_{i}=\frac{{TP}_{i}}{{TP}_{i}+{FP}_{i}},$$

(2) $${Precision}_{weighted}=\sum_{i=1}^{L}{Precision}_{i}\times w_{i},$$

(3) $${Recall}_{i}=\frac{{TP}_{i}}{{TP}_{i}+{FN}_{i}},$$

(4) $${Recall}_{weighted}=\sum_{i=1}^{L}{Recall}_{i}\times w_{i},$$

(5) $$Weighted\;F1=\frac{2\cdot {Precision}_{weighted}\cdot {Recall}_{weighted}}{{Precision}_{weighted}+{Recall}_{weighted}},$$

In this formula, i denotes the index of one individual class, with $w_{i}$ symbolizing the proportion of samples in that class relative to the overall sample count. L indicates the total number of classes. Additionally, TP is an abbreviation for true positives, FP means false positives, and FN designates false negatives.

In addition, we employed two other classic measurements: prediction accuracy (ACC) and Matthews correlation coefficient (MCC) [60,61]. ACC is defined as the proportion of correctly predicted samples, which is the commonly used measurement. However, when the dataset is imbalanced, ACC is not a perfect measurement. In this case, MCC is a better choice. This measurement is based on two matrices, *X* and *Y*, where *X* stores the true classes of samples and *Y* collects the predicted classes of all samples. Then, MCC can be computed by

(6) $$MCC=\frac{cov(X,Y)}{\sqrt{cov(X,X)\cdot cov(Y,Y)}},$$

where cov(X,Y) stands for the correlation coefficient of two matrices.

### 2.7. Functional Enrichment Analysis

Using the IFS method, we identified the most informative genes across various ranking methods. To elucidate the biological processes associated with genes in these subsets and their connection to heart conditions, we conducted gene ontology (GO) enrichment analysis. Additionally, the Kyoto Encyclopedia of Genes and Genomes (KEGG) pathway analysis was employed to pinpoint the relevant biological pathways. All these analyses are facilitated by the ClusterProfiler package in R (version 4.10.0) [62].

## 3. Results

**Figure 2:**
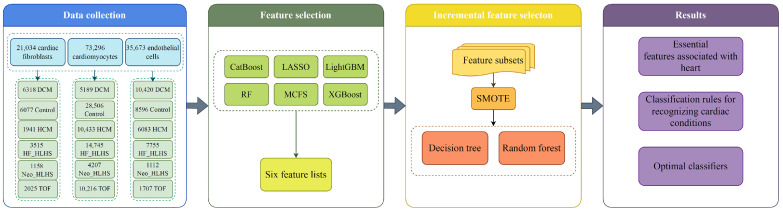
Flow chart of the entire analysis process. The single-cell data from 21,034 cardiac fibroblasts, 73,296 cardiomyocytes, and 35,673 endothelial cells are analyzed, which included six CHD forms: DCM, Control, HCM, HF_HLHS, Neo_HLHS, and TOF. Using six feature-ranking algorithms, six feature lists are generated. These lists are fed into the incremental feature selection framework. After above operations, essential features, classification rules, and optimal classifiers are obtained.

Our research provides a comprehensive analysis of the impact of gene expression on cardiac cells. We designed an analysis procedure that includes data collection, feature selection, IFS, and results. Figure 2 illustrates the detailed workflow. Using single-nucleus RNA sequencing data from Hill et al. ’s study [14], this study employs six feature-ranking algorithms to thoroughly evaluate feature (gene) significance. A pivotal element of our study is the utilization of the IFS method, pivotal in pinpointing key features for heart diseases. Our research culminates in the discovery of essential genes and quantitative classification rules. 

### 3.1. Feature Ranking Results

In this study, we employed six sophisticated feature-ranking algorithms—CatBoost, LASSO, LightGBM, MCFS, RF, and XGBoost—to meticulously analyze 29,266 gene features from 130,003 cardiac cells, including 21,034 CFs, 73,296 CMs, and 35,673 Endos. These approaches aimed to identify critical genes integral to heart health and disease. Each algorithm’s capability was harnessed to rank these genes based on their importance in a list, providing a nuanced understanding of their roles in cardiac function. The results are comprehensively presented in Table S1, offering a valuable resource for future cardiac research and potential clinical applications. For convenience, the lists yielded by six algorithms were called CatBoost, LASSO, LightGBM, MCFS, RF, and XGBoost feature lists.

### 3.2. IFS Results and Feature Intersections for Finding Key Features Associated with Heart

As each above-obtained feature list was very long, containing more than 20,000 features, the original IFS method, which added one feature one time, would need much time. Here, we set a step of five to reduce the possible feature subsets and only considered top 1000 features in each list. On each feature subset, two classifiers were built using DT and RF after samples were processed by SMOTE. All classifiers were evaluated by 10-fold cross-validation. The evaluation results are provided in Table S2. The weighted F1 score was selected as the key measurement for evaluating classifiers’ performance. To clearly show the performance of classifiers with the same classification algorithm and different top features, an IFS curve was plotted using the weighted F1 score as the Y-axis and the number of used features as the X-axis for DT or RF. Figures S1–S3 illustrate all IFS curves. 

**Figure 3:**
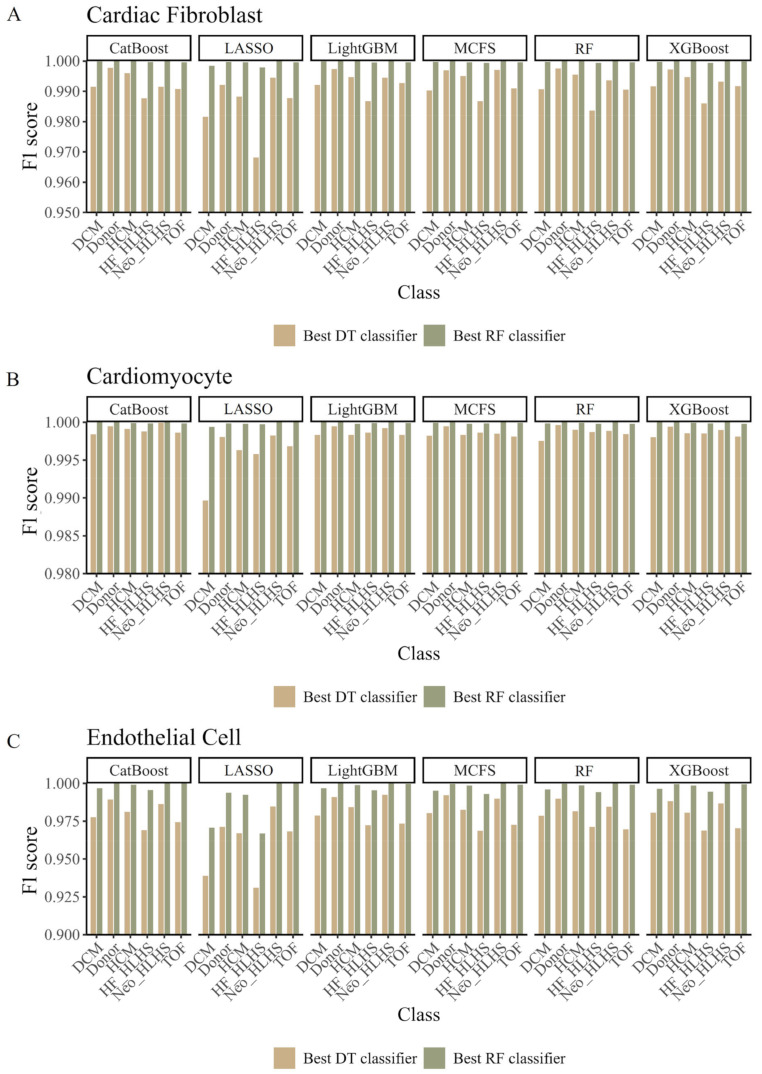
Performance of the best classifiers on six feature lists in six CHD classes. A grouped bar chart is utilized to compare the performance of two best classifiers based on random forest (RF) and decision tree (DT) between six CHD classes. (**A**): Grouped bar chart on CF data; (**B**): Grouped bar chart on CM data; (**C**): Grouped bar chart on Endo data.

For the IFS curves on CF (Figure S1), we can find that RF yielded the highest weighted F1 scores of 0.9998, 0.9989, 0.9998, 0.9996, 0.9997, and 0.9997 on six feature lists. Such performance was obtained by using the top 65, 305, 130, 335, 495, and 255 features in six lists. Accordingly, the best RF classifiers can be built using the above top features. Using the same arguments, the best DT classifiers can also be built, which used the top 150, 245, 370, 860, 220, and 535 features in six lists and yielded the weighted F1 scores of 0.9929, 0.9842, 0.9931, 0.9924, 0.9920, and 0.9926. The other measurements (ACC, MCC, and macro F1) of these best DT and RF classifiers are listed in Table 1, and their performance on six heart conditions classes is shown in Figure 3A. Among all the best classifiers, the RF classifiers using the top 65 features in the CatBoost feature list and the top 130 features in the LightGBM feature list provided the highest performance, with a weighted F1 score of 0.9998. They can be powerful tools to identify CHD conditions of CF cells. 

The IFS curves on CM are shown in Figure S2. When RF adopted the top 130, 100, 200, 310, 980, and 255 features in six feature lists, it yielded the highest weighted F1 scores of 0.9999, 0.9998, 0.9999, 0.9999, 0.9999, and 0.9999. As for DT, its highest performance was obtained by using the top 65, 940, 60, 105, 270, and 50 features in six lists. The weighted F1 scores were 0.9991, 0.9966, 0.9988, 0.9987, 0.9990, and 0.9988. Similarly, the best RF and DT classifiers can be built using the above features. Their detailed performance is shown in Table 1 and Figure 3B. Among all the best classifiers, the RF classifier using the top 255 features in the XGBoost feature list generated the highest performance, which can be a useful tool for predicting the CHD conditions of CM cells.

As for Endo, the IFS curves about this cardiac cell type are provided in Figure S3. The highest weighted F1 scores yielded by RF on six feature lists were 0.9976, 0.9812, 0.9976, 0.9965, 0.9971, and 0.9971. The top 190, 155, 165, 425, 250, and 215 features in six lists were used to produce the above performance. As for DT, it yielded the highest weighted F1 scores of 0.9791, 0.9524, 0.9812, 0.9808, 0.9797, and 0.9794 when the top 195, 335, 50, 40, 105, and 65 features in six lists were adopted. Likewise, the best DT and RF classifiers can be constructed. Table 1 and Figure 3C show their detailed performance. Among the constructed best classifiers, RF classifiers using the top 190 features in the CatBoost feature list and 165 features in the LightGBM feature list had the highest performance. They can be latent powerful tools for identifying CHD conditions of Endo cells.

It is interesting to compare the above optimal classifiers for CF, CM, and Endo with classifiers using all features. The 10-fold cross-validation results of the classifiers using all features are listed in Table 2. It was found that the performance of these classifiers is also very high. However, they were inferior to the optimal classifiers. This implied that through the feature-ranking algorithms and IFS procedure, essential features can be screened out, thereby improving the classifiers’ performance. 

**Figure 4:**
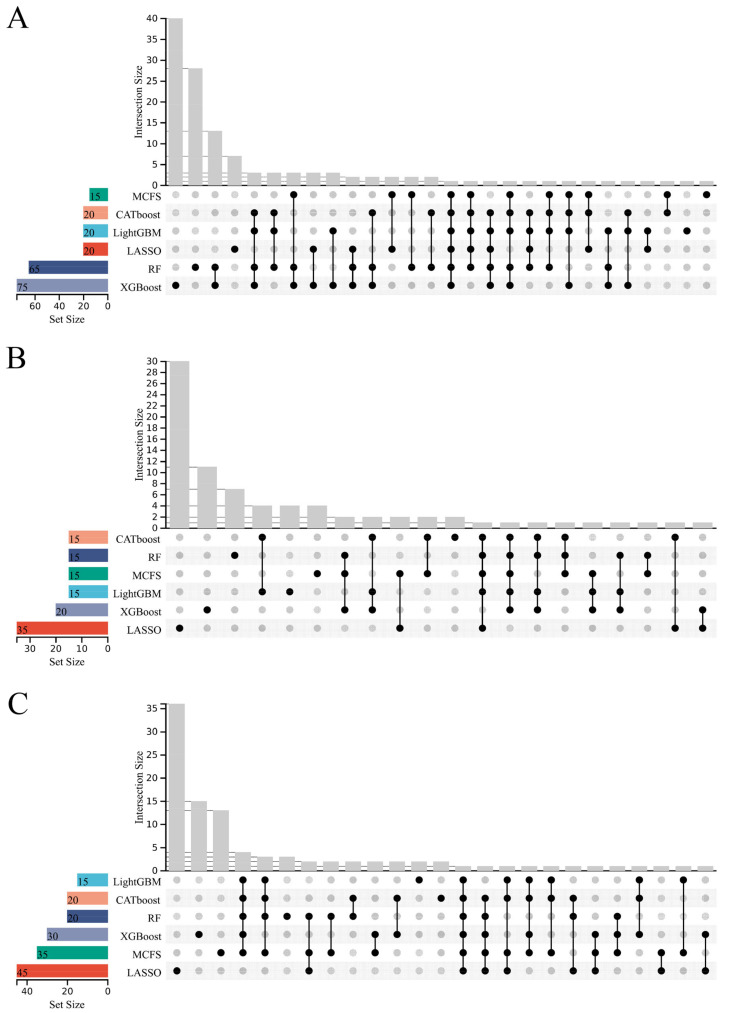
Upset graph of the essential feature subsets obtained using six feature-ranking algorithms. “Set Size” is the count of the number of features in each set; “Intersection Size” is the count of the number of features after taking the intersection of some feature sets; the black dots indicate the feature subsets identified by which feature-ranking algorithm; the line between the dots indicates the intersection of some feature subsets. (**A**): Upset graph on CF data; (**B**): Upset graph on CM data; (**C**): Upset graph on Endo data.

In Table 1, we can find that the best RF classifiers always provide better performance than the best DT classifiers. This meant that features used to construct these RF classifiers had a more powerful ability to isolate the CHD conditions of cardiac cells. The detailed analysis of these genes was helpful in uncovering the differences among cardiac cells with different CHD conditions. However, the numbers of these features for all best RF classifiers were large, inducing difficulties for detailed analysis. It was necessary to conduct further screening. By carefully checking the IFS results using RF, the RF classifier using much fewer features was accessed for each best RF classifier, which generated a little lower weighted F1 than the corresponding best RF classifier. We called these RF classifiers suboptimal RF classifiers. The performance of these classifiers is marked in Figures S1–S3 and Table 3. Clearly, these classifiers really adopted much fewer features, whereas their performance was slightly lower than the best RF classifiers. Evidently, features used in suboptimal classifiers were more essential than others used in the best classifiers. We selected these essential features for further investigation. For CF, the suboptimal RF classifiers used the top 20, 20, 20, 15, 65, and 75 features in six feature lists. For CM, the top 15, 35, 15, 15, 15, and 20 features in six lists were selected. For Endo, we selected the top 20, 45, 15, 35, 20, and 30 features in six lists. Accordingly, six essential feature groups were obtained for each cardiac cell type. These groups were identified by six different feature-ranking algorithms. An upset graph was plotted to show the differences and commons among these groups for each cardiac cell type, as shown in Figure 4. It can be observed that some features (genes) were in multiple groups, indicating that they were identified to be essential by multiple feature-ranking algorithms. For example, *XIST* was in all six groups for CF and Endo; *ARHGAP24* and *FKBP5* were in five groups for CM. Table S3 lists the detailed genes in one or more groups. In Section 4, some identified genes will be discussed. 

### 3.3. Establishing Classification Rules for Identifying Congenital Heart Diseases

**Figure 5:**
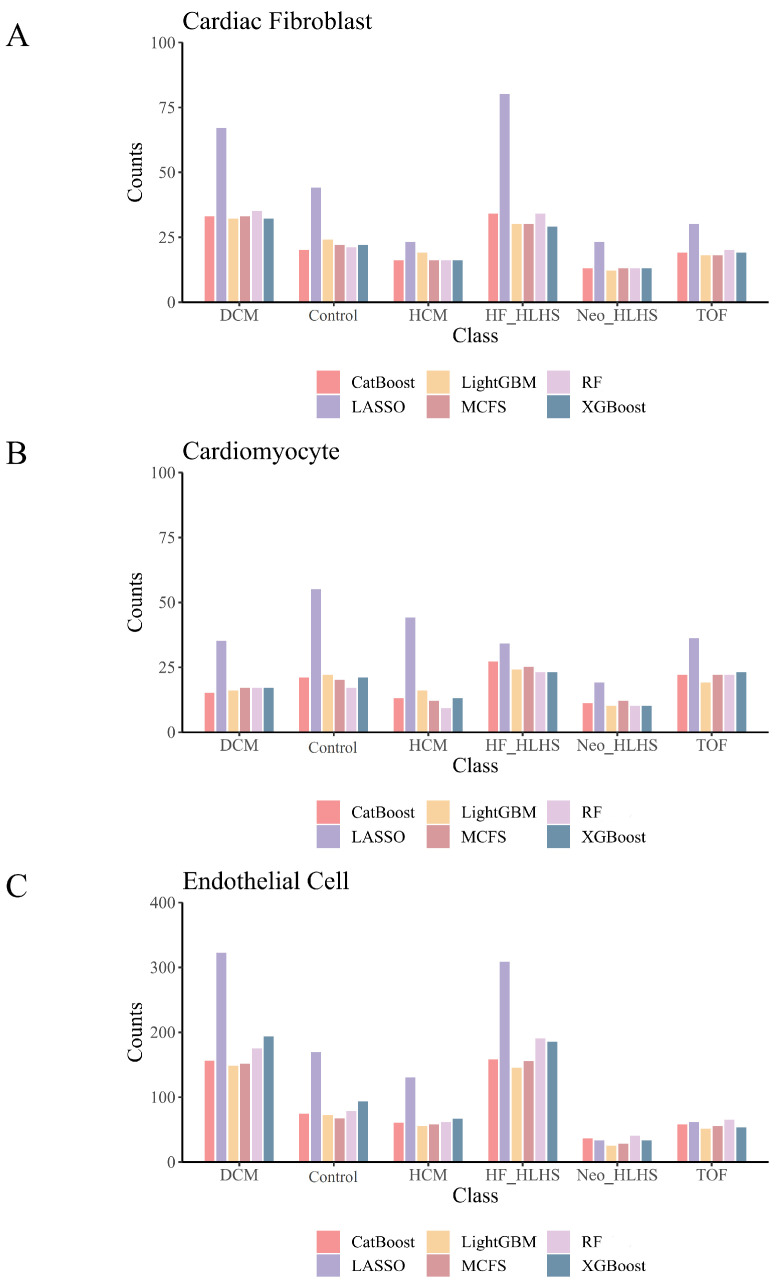
Bar plot showing the number of rules for identifying CHD condition classes. For six classes, the number of rules extracted from the best DT classifiers on six feature lists is shown. The different colors represent the different feature lists obtained by six feature-ranking algorithms. (**A**): Bar plot on CF data; (**B**): Bar plot on CM data; (**C**): Bar plot on Endo data.

Except for the discovery of essential genes, we also tried to explore different expression patterns of six CHD conditions for each cardiac cell type. This task was completed by DT. In Section 3.2, the best DT classifier was built on each feature list for each cardiac cell type. From this DT classifier, a rule group can be obtained. Tables S4–S6 comprehensively lists these rules. In each rule group, some groups were in charge of classifying cells into corresponding CHD condition classes. Figure 5 depicts the number of rules across each class. On the one hand, these rules can be used to predict various CHD conditions, including DCM, controls, HCM, HF_HLHS, Neo_HLHS, and TOF. On the other hand, each rule displayed a special expression pattern, represented by some genes and their expression level thresholds, on one CHD condition that was the result of the rule. Section 4 delves into the significance of these genetic rules and their implications for understanding CHD.

### 3.4. Enrichment Analysis for Essential Genes

**Figure 6:**
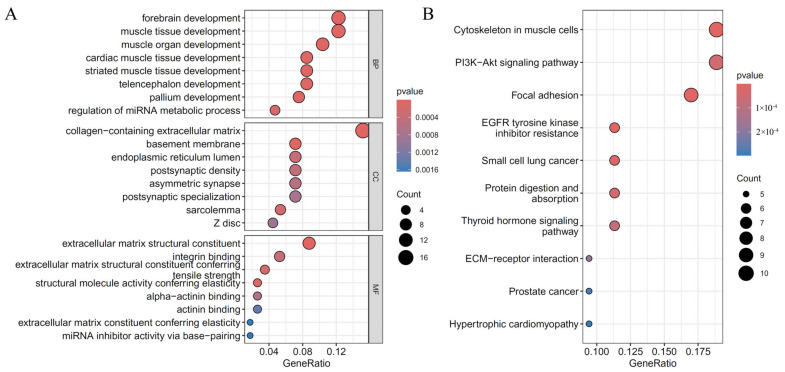
Results of the functional enrichment analysis on essential genes in the cardiac fibroblasts. (**A**): Results on GO terms; (**B**): Results on KEGG pathways.

**Figure 7:**
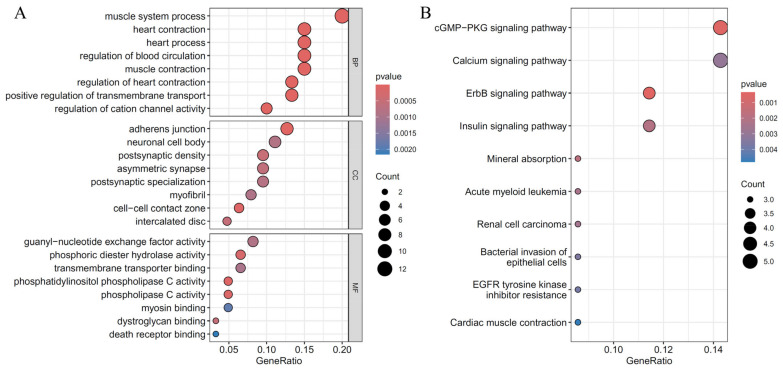
Results of the functional enrichment analysis on essential genes in the cardiomyocytes. (**A**): Results on GO terms; (**B**): Results on KEGG pathways.

**Figure 8:**
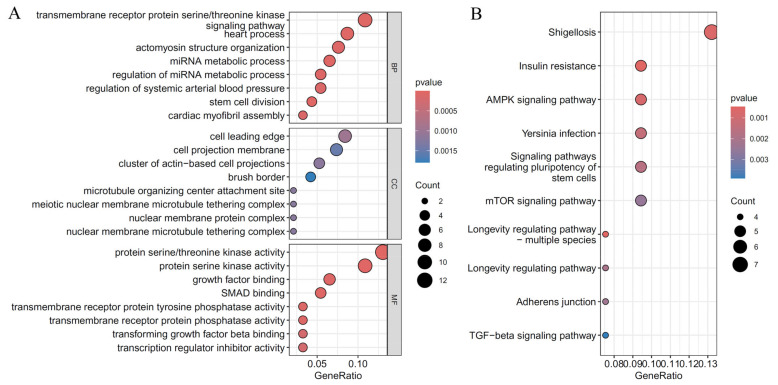
Results of the functional enrichment analysis on essential genes in the endothelial cells. (**A**): Results on GO terms; (**B**): Results on KEGG pathways.

As mentioned above, the essential genes were defined as those used to construct suboptimal RF classifiers. For each cardiac cell type, six essential gene groups were combined. Then, the functional enrichment analysis was conducted on these combined genes to uncover the biological meanings behind these genes. Figure 6, Figure 7 and Figure 8 display the top GO terms in three clusters: biological processes (BP); cellular components (CC); molecular functions (MF); and top KEGG pathways for CF, CM, and Endo. 

For CF, significant GO terms associated with CHD included “muscle tissue development,” “cardiac muscle tissue development,” “extracellular matrix structural constituent,” and “integrin binding,” and pathways such as “Hypertrophic cardiomyopathy,” “ECM-receptor interaction,” and “Focal adhesion.” (Figure 6) These findings highlight the crucial roles of muscle development, extracellular matrix (ECM) integrity, and ECM-cell interactions in cardiac fibroblast function and CHD pathogenesis. For CM, significant GO terms associated with CHD included the “muscle system process” and “heart contraction,” and pathways including “calcium signaling pathway” and “cGMP-PKG signaling pathway.” (Figure 7) These findings emphasize the critical roles of muscle contraction and key signaling processes in cardiomyocyte function and CHD pathogenesis. Understanding these enriched outcomes offers valuable insights into potential therapeutic targets for CHD. For Endo, significant GO terms associated with CHD included “transmembrane receptor protein serine/threonine kinase signaling pathway,” “actomyosin structure organization”, and “regulation of miRNA metabolic process,” and pathways including “AMPK signaling pathway,” and “TGF-beta signaling pathway.” (Figure 8) These findings underscore the importance of cell signaling, structural organization, and gene regulation in endothelial cell function and CHD pathogenesis. Understanding these enriched pathways provides insights into potential therapeutic targets for CHD.

These enriched GO terms and KEGG pathways for each cell type provide valuable insights into the BPs, CCs, and MFs associated with heart conditions. This comprehensive analysis enhances the understanding of genetic factors influencing cardiac health, offering a foundation for further research in the field. 

## 4. Discussion

Here, in this study, we utilized a series of effective machine learning models, including CatBoost, RF, MCFS, LightGBM, XGBoost, and LASSO, for optimized feature selection and classification modelling. Significant cell-type level transcriptomic features and quantitative rules were identified to distinguish cell-type expression profiling for different forms of CHD. The identified genes and rules can not only reveal molecular patterns of different CHD subtypes in three common cell types, including cardiomyocytes, cardiac fibroblast, and endothelial cells, but also provide us a reproducible approach to evaluate and screen cell-type specific molecular gene signatures for complex diseases with potential subtypes. A detailed discussion is presented below, showing cell-type-specific regulatory mechanisms for different CHD subtypes.

### 4.1. Optimized Features Selected by LASSO

We identified up-regulation of gene *FOXO3* (Forkhead Box O3, 602681) in cardiac fibroblasts, which can further help us identify dilated cardiomyopathy. Such a finding has been validated by German scientists [63]. Down-regulation of *AGAP1* (ArfGAP With GTPase Domain, Ankyrin Repeat, and PH Domain 1, 608651) in neo-hypoplastic left heart syndrome and up-regulation of such a gene in HF-hypoplastic left heart syndrome revealed the different effects of such gene during the pathogenesis of different CHD subtypes [64]. As for Tetralogy of Fallot, *SHROOM3* (Shroom Family Member 3, 604570) as the gene signature for such CHD subtypes has been shown to be functionally related to CHD according to a previous study [65]. Using the LASSO module in cardiomyocyte transcriptomics also identified a series of specific gene signatures for quantitative CHD subtyping. *TMTC1* (Transmembrane O-Mannosyltransferase Targeting Cadherins 1, 615855), as a specific gene signature in our rules for dilated cardiomyopathy identification, is shown to be associated with the risk of heart failure [66], which is a common symptom for such CHD subtype [67]. A common variants analysis of complex diseases revealed that *ARHGAP24* (Rho GTPase Activating Protein 24, 610586)-associated variants are functionally associated with hypertrophic cardiomyopathy [68], validating our predicted rules. Similarly, such a gene has also been shown to be predictive for HF-hypoplastic left heart syndrome with a lower expression level, which can also be validated by such publications [68]. Although no direct reports confirmed the association between *XIST* (X Inactive Specific Transcript, 300936) and Tetralogy of Fallot, abnormal *XIST* (X Inactive Specific Transcript) expression has been widely observed in different CHD subtypes [69,70]. The identification of *XIST* (X Inactive Specific Transcript) as a potential gene signature validated the efficacy and accuracy of our prediction. Endothelial cell-based prediction identified a series of different literature-supported gene signatures like *COL25A1* (Collagen Type XXV Alpha 1 Chain, 610004) for dilated cardiomyopathy [71] and neo/HF-hypoplastic left heart syndrome [72], *NFIB* (Nuclear Factor I B, 600728) for hypertrophic cardiomyopathy [73], and *KLF7* (Kruppel-Like Transcript Factor 7, 604865) for Tetralogy of Fallot [74]. We further compared the prediction results from other machine learning model methods, as presented below. 

### 4.2. Optimized Features Selected by LightGBM

Different from the prediction result of LASSO, in the cardiac fibroblasts, we recognized the up-regulation of *ARL15* for dilated cardiomyopathy, which has already been reported previously [75]. *ARL15* (ADP Ribosylation Factor Like GTPase 15, 604699) mediated the effects of the catecholamine-β-adrenoceptor-cAMP system for such CHD subtype [75]. Here, based on LightGBM, we utilized *XIST* (X Inactive Specific Transcript, 300936) as an effective gene signature to identify both hypertrophic cardioMyopathy (up-regulation) and HF-hypoplastic left heart syndrome (down-regulation), specifically in fibroblasts, which have already been supported by previous publications [69,70]. Variants of *PTEN* (Phosphatase And Tensin Homolog, 601728), another transcriptomic gene signature we identified from our quantitative rules, have also been confirmed to play an important role in neo-hypoplastic left heart syndrome, validating our prediction [76]. Cardiomyocytes find different gene signatures for CHD subtype prediction. *FKBP5* (FKBP Prolyl Isomerase 5, 602623) was shown to be down-regulated in dilated cardiomyopathy, as reported by previous transcriptomic and methylation studies [77,78,79,80]. *PSD3* (Pleckstrin And Sec7 Domain Containing 3, 614440) has been shown to contain specific functional copy number variants at the genomic level during the pathogenesis of Neo/HF-hypoplastic left heart syndrome [81]. In endothelial cells, as we have discussed above, *COL25A1* (Collagen Type XXV Alpha 1 Chain, 610004) is shown to be an effective gene signature for dilated cardiomyopathy [71], also predicted by LightGBM. As for Tetralogy of Fallot, PDE4D (Phosphodiesterase 4D, 600129) is a widely identified heart disease-associated gene signature and has been reported to be associated with different subtypes of CHD in endothelial cells, including Tetralogy of Fallot [82,83].

### 4.3. Optimized Features Selected by CatBoost

As we have discussed above, the specific role of *ARL15* (ADP Ribosylation Factor Like GTPase 15, 604699) in dilated cardiomyopathy prediction, *XIST* (X Inactive Specific Transcript) for hypertrophic cardiomyopathy, and hypoplastic left heart syndrome has been validated. The consistent prediction results of CatBoost with other prediction models in cardiac fibroblasts validate the efficacy of using machine learning models for cell-type and disease-subtype-specific expression profiling predictions. *PTEN* (Phosphatase And Tensin Homolog) was further predicted to identify hypoplastic left heart syndrome with a higher expression level. According to recent publications, such expression character has been validated [76,84]. The associations between the Tetralogy of Fallot and the predicted gene *CRISPLD2* (Cysteine Rich Secretory Protein LCCL Domain Containing 2) have also been validated by a systemic analysis for the pathogenesis of right ventricular (RV) failure [85]. The specific role of *XIST* (X Inactive Specific Transcript) in HF-hypoplastic left heart syndrome has been reported to be associated with the specific inactivation of the ring X chromosome [86]. *PDE3A* (Phosphodiesterase 3A, 123805) together with *PDE4* (Phosphodiesterase 4A, 600129) has been shown to control the intracellular cAMP and cardiac excitation–contraction coupling, which were further confirmed to be associated with Tetralogy of Fallot [82], validating our prediction. As for endothelial cells, similarly to the discussion above, associations between *EMCN* and hypertrophic cardiomyopathy have been fully validated [87,88]. The HF-hypoplastic left heart syndrome can be easily identified using *FKBP5* (FKBP prolyl isomerase 5, 602623), a gene that we have discussed above to be associated with such CHD subtype, while, at the same time, the neo-hypoplastic left heart syndrome can be predicted by EMP1, for which the associations between which two were further supported by a recent publication [89]. 

### 4.4. Optimized Features Selected by MCFS

MCFS identified a series of common gene signatures in cardiac fibroblasts, which we have discussed above, like *ARL15* in dilated cardiomyopathy. Apart from that, we also recognized the down-regulation of *COL1A2* (Collagen Type I Alpha 2 Chain, 120160) for neo-hypoplastic left heart syndrome, specifically in fibroblast and macrophage [90]. As for another gene, *TACC1* (Transforming Acidic Coiled-Coil Containing Protein 1, 605301), it has been shown to be associated with CHD, as we have discussed above, but the association between such gene with HF-hypoplastic left heart syndrome has not been detailed and confirmed. In cardiomyocytes, we also observed a low expression level of *FKBP5* (FKBP Prolyl Isomerase 5, 602623), just like we have seen in the prediction results of other methods. As for hypertrophic cardiomyopathy, *PSD3* (Pleckstrin And Sec7 Domain Containing 3, 614440), as a pathogenic gene for Idiopathic Peripheral Autonomic Neuropathy, has also been reported to be differentially expressed across different CHD subtypes, validating our prediction [91]. MCFS also identified a series of endothelial cell-based gene signatures for CHD subtyping, which are consistent with the results of other methods. Interestingly, *EMCN* (Endomucin, 608350), which has previously been shown to be associated with hypertrophic cardiomyopathy, was predicted to be associated with dilated cardiomyopathy using such a method, supported by another publication [92]. The up-regulation of *XIST* in hypertrophic cardiomyopathy has also been predicted by other methods like LightGBM and CatBoost and functionally validated by several publications [69,70]. Gene signatures like *COL25A1* (Collagen Type XXV Alpha 1 Chain, 610004) for hypoplastic left heart syndrome neo hypoplastic left heart syndrome and *FKBP5* (FKBP Prolyl Isomerase 5, 602623) for HF-hypoplastic left heart syndrome have also been discussed above. As for Tetralogy of Fallot, *NFIB* (nuclear factor I B, 600728) was shown to be associated with such CHD subtype in infants through microRNA regulation [93], validating the efficacy and accuracy of our prediction.

### 4.5. Optimized Features Selected by RF

The RF model is a classic machine learning model for disease subtyping and gene signature identification. A series of gene signatures identified in cardiac fibroblasts have already been discussed above and validated to be effective by recent publications. *ARL15* (ADP Ribosylation Factor Like GTPase 15, 604699) has been widely shown to be associated with dilated cardiomyopathy by multiple machine learning models with reliable literature support [75]. Some new genes identified for RF-based CHD subtyping in cardiac fibroblasts have also been supported by recent publications like *USP53* (Ubiquitin Specific Peptidase 53, 617431) in hypertrophic cardiomyopathy [94], *CUX1* (cut like homeobox 1, 116896) for neo-hypoplastic left heart syndrome [95], and *BAI3* (Brain-Specific Angiogenesis Inhibitor 3, 602684) for Tetralogy of Fallot [96]. As for the cardiomyocytes, *FKBP5* (FKBP Prolyl Isomerase 5, 602623) had previously been reported to be down-regulated in dilated cardiomyopathy [77,78,79,80]. Shared gene signatures *FRMD5* (FERM Domain Containing 5, 616309)) were shown to be associated with neo/HF-hypoplastic left heart syndrome but with different directions predicted by various prediction methods [97]. *FILIP1L* (Filamin A Interacting Protein 1 Like, 612993), as a component of the protein filamin, has also been shown to be associated with nonsyndromic Tetralogy of Fallot in an Iranian family based on a comprehensive whole-exome analysis [98], consistent with our prediction on such gene as a gene signature. In the endothelial cell, *EMCN* (Endomuci, 608350) has been widely discussed above to be associated with different CHD subtypes, including dilated cardiomyopathy, validating our prediction. Using LightGBM, we have already identified *PDE4D* (Phosphodiesterase 4D, 600129) as a specific gene signature for the Tetralogy of Fallot [82,83]. Here, using a random forest algorithm, we validated these results and further confirmed the specific role of *PDE4D* (Phosphodiesterase 4D, 600129) in such a CHD subtype.

### 4.6. Optimized Features Selected by XGBoost

As the final method for CHD subtype single-cell-type-level analysis, the top results in cardiac fibroblasts have mostly also been identified by LightGBM and RF. We identified the down-regulation of HIF3A for dilated cardiomyopathy, *SCN7A* (Sodium Voltage-Gated Channel Alpha Subunit 7, 182392) for hypertrophic cardiomyopathy, *MALAT1* (Metastasis Associated Lung Adenocarcinoma Transcript 1) up-regulation for HF-hypoplastic left heart syndrome, and down-regulation for neo-hypoplastic left heart syndrome, which have been confirmed by previous publications [99,100,101]. For Tetralogy of Fallot, XGBoost identified a specific gene signature *ZEB1* (zinc finger E-box binding homeobox 1, 189909), which has also been validated by a recent publication [102]. As for cardiomyocytes, we have identified EDA as an effective gene signature for dilated cardiomyopathy. *FKBP5* (FKBP prolyl isomerase 5, 602623) is associated with various subtypes of CHD, according to our discussion above. It has also been shown to be up-regulated in hypertrophic cardiomyopathy, as previously reported [103], associated with heart failure. *EMCN* (Endomucin), as we have discussed above for the prediction results from different machine learning models, has been shown to be associated with various CHD subtypes, including dilated cardiomyopathy, with literature support [92]. ITGA1(Integrin Subunit Alpha 1) has been shown to be associated with marginal zinc deficiency in heart tissue in vivo [104], which leads to the initiation and progression of hypertrophic cardiomyopathy, validating our predictions. *MYH7* (Myosin Heavy Chain 7, 160760), as the gene signature we predicted together with its homolog, has been identified from a genetic analysis of HF-hypoplastic left heart syndrome, revealing the potential pathogenic effect of such a gene. As for neo-hypoplastic left heart syndrome, *PIK3R3* (Phosphoinositide-3-kinase Regulatory Subunit 3, 606076) has been shown to alter the metabolic and inflammatory metabolism in the heart of neonates with congenital heart disease [105]. 

All in all, as we have discussed above, we utilized cell-type specific expression profiling to distinguish different CHD subtypes, including Tetralogy of Fallot (TOF), hypoplastic left heart syndrome (HLHS, including Neo-HLHS and HF-HLHS), hypertrophic cardiomyopathy (HCM), and dilated cardiomyopathy, comparing with donors’ control population. The identified quantitative rules with significant transcriptomic features in different cell types can help us understand the complex cellular microenvironment during CHD pathogenesis and reveal cell-type specific driven mechanisms for different CHD subtypes. Interestingly, identified gene signatures not only show specific expression profiling across different CHD subtypes but also have been reported to include pathological functional variants, indicating that the identified genes may contribute to CHD pathogenesis across different omics levels.

### 4.7. Functional Analysis of the Key Features of CHD

In analyzing key CHD genes in cardiac fibroblasts, significant enrichment results are linked to muscle tissue development and extracellular matrix (ECM) components. GO terms such as “forebrain development,” “muscle tissue development,” and “cardiac muscle tissue development” underscore the importance of these developmental processes in the pathogenicity of CHD. A disruption in these results, for example, by mutations in *MYH7* and *TTN*, results in cardiomyopathies [80,106]. Another condition that affects this pathway is structural heart defects such as hypoplastic left heart syndrome and dilated cardiomyopathy. GO terms enriched in the categories “extracellular matrix structural constituent” and “integrin binding” point to the importance of cell adhesions and the extracellular matrix. The ECM maintains structural support and regulates the behavior of cells. Abnormal development and functioning of the heart that results from mutations in ECM-related genes may lead to dilated cardiomyopathy and hypoplastic left heart syndrome, such as *COL25A1* or *COL1A2* [71,90].

In analyzing key CHD genes in cardiomyocytes, significant enrichment results highlight muscle system processes and key signaling pathways. GO terms such as “muscle system process,” “heart contraction,” and “regulation of heart contraction” underscore their critical role in CHD pathogenesis. Disruptions in these findings, due to mutations in genes like *MYL4* and *TTN*, lead to cardiomyopathies and structural defects [80,106]. Additionally, enriched KEGG pathways like “calcium signaling” and “cGMP-PKG signaling” emphasize their importance in cardiomyocyte function, with genes such as *TRPM7* and *PIK3R1* playing crucial roles [107,108]. Understanding these results and their gene associations provides insights into potential therapeutic targets for CHD.

The significant GO results represented in the dataset for the endothelial cells include the “transmembrane receptor protein serine/threonine kinase signaling pathway,” “actomyosin structure organization,” and “regulation of miRNA metabolic process.” These all point to the role of cell signaling, structural organization, and gene regulation in an endothelial cell that could lead to CHD. Crucial genes that participate in these results are *COL25A1* and *TTN*, which are essential for structural integrity and signaling in the endothelial cells [71,80]. KEGG pathways, such as “The AMPK signaling pathway” and “The TGF-beta signaling pathway,” with genes like *SMAD6* and *AKT3*, respectively, indicate functions in cellular metabolism and growth regulation [109,110]. However, they further hint at their involvement in CHD: the enriched pathways and the respective genes help one understand a possible target therapy implicated in CHD.

### 4.8. Redundancy of Predicted Genes across Different Congenital Heart Disease Subtypes

As we have discussed above, we recognized various quantitative rules, including functional gene signatures associated with different congenital heart disease subtypes. Although shared genes like *TMTC1, ART3, and ARHGAP24* are shown to contribute to different congenital heart disease subtypes, they actually play different roles across subtypes. For instance, as we have discussed above, *ARHGAP24* contributes to various subtypes of congenital heart disease, including dilated cardiomyopathy, hypertrophic cardiomyopathy, and HF-hypoplastic left heart syndrome. However, *ARHGAP24* plays different roles during the pathogenesis of three subtypes of congenital heart diseases. *ARHGAP24* is up-regulated in hypertrophic cardiomyopathy while down-regulated in HF-hypoplastic left heart syndrome. Considering that *ARHGAP24* can participate in the rho GTPase-activating process, different roles of *ARHGAP24* across different congenital heart diseases indicate different roles of rho GTPase activation across different disease subtypes. Similarly, *XIST* has also been recognized to be associated with various congenital heart disease subtypes. *XIST* has been widely reported to participate in congenital heart disease through various lncRNAs-miRNAs regulation [70]. Different microRNAs and lincRNAs like miR-27a-3p, miR-130b-3p, Jpx, and Xist have been shown to participate in the initiation and progression of different subtypes of congenital heart diseases [70]. For dilated cardiomyopathy, *XIST* acts with *IDI2-AS1* together to contribute to disease pathogenesis [111], while for hypertrophic cardiomyopathy, XIST acts with miR-330-3p [112,113], validating that different pathogenic mechanisms are involved even for the same genes across different congenital heart disease subtypes.

### 4.9. Variants and Expression Profiling Congenital Heart Disease Subtyping

As we have discussed above, we utilized expression profiling to recognize specific molecular signatures for different congenital heart disease subtypes. In our discussion, we also recognized various genetic effects on predicted genes, implying the complex multi-Omic-level regulatory effects on congenital heart disease pathogenesis. For instance, genetic variants on *ARHGAP24* have been recognized as contributing to the pathogenesis of hypertrophic cardiomyopathy [68]. Apart from *ARHGAP24*, *PTEN*, as another predicted gene contributing to congenital heart disease, has also been mentioned to contribute to neohypoplastic left heart syndrome at both transcriptomics (gene expression profiling, as shown in this study) and genetics levels [76]. Similarly, PSD3 (Pleckstrin And Sec7 Domain Containing 3) has been shown to have specific copy number variants for CHD subtypes, including neo/HF-hypoplastic left heart syndrome [81], validating the specific role of genetic variants on congenital heart disease subtyping. Therefore, the molecular differences across congenital heart disease subtypes have been recognized at the multi-Omics level, with both transcriptomic differences and genetic variants, implying the complexity of congenital heart disease pathogenesis.

Genetic variants and expression profiling alterations reflect two different but highly connected molecular entities. Following the center dogma system, genetic variants initiated the abnormal molecular alterations following the variations in sequences. Transpassing through transcription, transcription regulation, and post-transcription regulation, the genetic variants can induce specific gene expression alterations either at mRNA or protein levels. The combination of genetic variants and gene expression profiling provides a comprehensive understanding of the molecular changes of disease and reveals the internal heterogeneity of disease like CHD. Computationally, approaches like quantitative trait loci analyses, transcriptome-wide association analyses, proteome-wide association analyses, and colocalization analyses connect genetic variants functioning with expression alterations, providing us with a series of comprehensive approaches for disease pathogenesis exploration.

### 4.10. Comparison of the Public CHD-Related Genes

**Figure 9:**
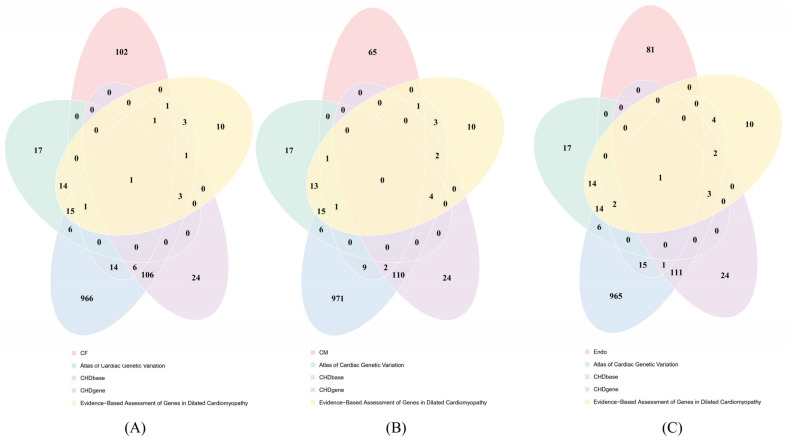
Venn diagram to show the commons and differences among CHD essential genes derived from three cell types and public CHD-related genes in four resources (Atlas of Cardiac Genetic Variation, CHDbase, CHDgene, an article titled by Evidence-Based Assessment of Genes in dilated cardiomyopathy). (**A**) Venn diagram for cardiac fibroblast (CF); (**B**) Venn diagram for cardiomyocytes (CM); (**C**) Venn diagram for endothelial cells (Endo). CHD essential genes derived from each cell type have common genes in four public resources.

To validate the reliability of the genes reported in this study, we employed the CHD-related genes reported in three public resources: Atlas of Cardiac Genetic Variation (https://www.cardiodb.org/acgv/index.php, accessed on 5 July 2024) [114], CHDbase (http://chddb.fwgenetics.org/, accessed on 5 July 2024) [115], and CHDgene (https://chdgene.victorchang.edu.au/, accessed on 5 July 2024) [116], and one published article [117]. The above genes constituted four CHD-related gene groups. For the discovered genes from CF, CM, and Endo, Venn diagrams were plotted to show the commons and differences among five gene groups, as shown in Figure 9. It can be observed that the CHD essential genes derived from three cell types (CF, CM, and Endo) all have common genes with those collected in the above CHD-related gene groups, suggesting the reliability of the obtained CHD essential genes in this study.

### 4.11. Limitations

The major concern for current analyses is the data resources for disease clustering. Hsieh et al.’s data represent the premature status of the human heart during the development processes, but not the pathological condition of the human heart. Since it is really hard to obtain the developed samples, our premature data reflect the original pathological background of different CHD subtypes, connecting genes with pathological potentials. Apart from that, clinically, it is almost impossible to collect different cell type samples from different CHD subtypes. The utilization of premature samples provides a reliable approach to disease pathogenesis with fewer medical ethics issues. 

However, our group also admitted that limitations exist in this study. The premature samples cannot 100% reflect the pathogenesis of different CHD subtypes in real time. In this study, we established a methodological platform that allows us to recognize disease subtype molecular signatures through a machine learning-based computational approach. Methodologically, we have overcome the obstacle of disease subtype-related gene signature recognition. Further collections on samples directly from patients’ hearts can help us establish a more accurate molecular profling of the complex disease, CHD.

## 5. Conclusions

In this study, we accomplished both a methodological improvement and a biological exploration. We established a comprehensive single-cell sequencing and machine learning integrated approach to analyze gene expression profiles and recognize specific disease related biomarkers quantitatively and qualitatively. The computational approach is a general platform for complex disease pathogenesis exploration. Apart from that, biologically, based on a reliable premature data cohort, we recognized single-cell-level gene biomarkers for different CHD subtypes. Models like LASSO, LightGBM, and CatBoost highlighted genes such as *FOXO3* and *TMTC1* as potential gene signatures for various CHD forms. Our findings not only demonstrate the efficacy of machine learning in identifying CHD-specific gene signatures but also develop quantitative rules for representing the gene expression patterns associated with CHDs. This research highlights the potential of machine learning in decoding the molecular complexities of CHD and lays a solid foundation for future mechanism-based studies in this field. The codes are available at https://github.com/chenlei1982/CHD_SingleCell.

## Figures and Tables

**Table 1:** Performance of the optimal classifiers for three cardiac cell types.

Cell Type	Feature-Ranking Algorithm	Classification Algorithm	Number of Features	ACC	MCC	Macro F1	Weighted F1
CF	CatBoost	RF	65	0.9998	0.9997	0.9998	0.9998
		DT	150	0.9929	0.9909	0.9924	0.9929
	LASSO	RF	305	0.9989	0.9986	0.9991	0.9989
		DT	245	0.9842	0.9797	0.9853	0.9842
	LightGBM	RF	130	0.9998	0.9997	0.9998	0.9998
		DT	370	0.9931	0.9911	0.9929	0.9931
	MCFS	RF	335	0.9996	0.9995	0.9996	0.9996
		DT	860	0.9924	0.9902	0.9927	0.9924
	RF	RF	495	0.9997	0.9996	0.9997	0.9997
		DT	220	0.9920	0.9897	0.9918	0.9920
	XGBoost	RF	255	0.9997	0.9996	0.9998	0.9997
		DT	535	0.9926	0.9905	0.9923	0.9926
CM	CatBoost	RF	130	0.9999	0.9999	0.9999	0.9999
		DT	65	0.9991	0.9988	0.9990	0.9991
	LASSO	RF	100	0.9998	0.9997	0.9998	0.9998
		DT	940	0.9965	0.9955	0.9958	0.9966
	LightGBM	RF	200	0.9999	0.9999	0.9999	0.9999
		DT	60	0.9988	0.9985	0.9987	0.9988
	MCFS	RF	310	0.9999	0.9999	0.9999	0.9999
		DT	105	0.9987	0.9983	0.9985	0.9987
	RF	RF	980	0.9999	0.9998	0.9999	0.9999
		DT	270	0.9989	0.9986	0.9986	0.9990
	XGBoost	RF	255	0.9999	0.9999	0.9999	0.9999
		DT	50	0.9988	0.9984	0.9986	0.9988
Endo	CatBoost	RF	190	0.9976	0.9970	0.9983	0.9976
		DT	195	0.9791	0.9731	0.9795	0.9791
	LASSO	RF	155	0.9812	0.9758	0.9871	0.9812
		DT	335	0.9524	0.9388	0.9600	0.9524
	LightGBM	RF	165	0.9976	0.9970	0.9983	0.9976
		DT	50	0.9812	0.9758	0.9818	0.9812
	MCFS	RF	425	0.9965	0.9955	0.9974	0.9965
		DT	40	0.9807	0.9752	0.9808	0.9808
	RF	RF	250	0.9971	0.9962	0.9978	0.9971
		DT	105	0.9797	0.9739	0.9790	0.9797
	XGBoost	RF	215	0.9971	0.9963	0.9979	0.9971
		DT	65	0.9794	0.9735	0.9791	0.9794

**Table 2:** Performance of the RF classifiers using all features for three cardiac cell types.

Cell Type	ACC	MCC	Macro F1	Weighted F1
CF	0.9976	0.9969	0.9978	0.9976
CM	0.9996	0.9995	0.9996	0.9996
Endo	0.9796	0.9740	0.9850	0.9796

**Table 3:** Performance of the suboptimal classifiers for three cardiac cell types.

Cell Type	Feature-Ranking Algorithm	Classification Algorithm	Number of Features	ACC	MCC	Macro F1	Weighted F1
CF	CatBoost	RF	20	0.9990	0.9987	0.9991	0.9990
	LASSO	RF	20	0.9929	0.9909	0.9942	0.9929
	LightGBM	RF	20	0.9992	0.9990	0.9991	0.9992
	MCFS	RF	15	0.9927	0.9906	0.9940	0.9927
	RF	RF	65	0.9990	0.9988	0.9992	0.9991
	XGBoost	RF	75	0.9991	0.9989	0.9992	0.9991
CM	CatBoost	RF	15	0.9995	0.9994	0.9995	0.9995
	LASSO	RF	35	0.9992	0.9989	0.9991	0.9992
	LightGBM	RF	15	0.9996	0.9994	0.9996	0.9996
	MCFS	RF	15	0.9993	0.9990	0.9992	0.9993
	RF	RF	15	0.9992	0.9990	0.9991	0.9992
	XGBoost	RF	20	0.9993	0.9991	0.9993	0.9993
Endo	CatBoost	RF	20	0.9921	0.9898	0.9927	0.9921
	LASSO	RF	45	0.9723	0.9645	0.9805	0.9724
	LightGBM	RF	15	0.9903	0.9875	0.9914	0.9903
	MCFS	RF	35	0.9917	0.9894	0.9934	0.9917
	RF	RF	20	0.9904	0.9876	0.9915	0.9904
	XGBoost	RF	30	0.9900	0.9872	0.9915	0.9900

## Data Availability

The data presented in this study are openly available in Hill et al.’s study, reference number [14].

## Supplementary Materials

The following supporting information can be downloaded at: https://www.mdpi.com/article/10.3390/life14081032/s1, Table S1: Feature ranking results obtained using CatBoost, LASSO, LightGBM, MCFS, RF, and XGBoost. Table S2: IFS results with two different classification algorithms on CatBoost, LASSO, LightGBM, MCFS, RF, and XGBoost feature lists. Table S3: Intersection of six essential gene sets extracted from the CatBoost, LASSO, LightGBM, MCFS, RF, and XGBoost feature lists. Table S4: Classification rules generated by decision tree using its optimal features on six feature lists for cardiac fibroblasts. Table S5: Classification rules generated by decision tree using its optimal features on six feature lists for cardiomyocytes. Table S6: Classification rules generated by decision tree using its optimal features on six feature lists for endothelial cells. Figure S1: IFS curves for evaluating the performance of two classification algorithms based on the weighted F1 score in cardiac fibroblasts. A: IFS curves on CatBoost feature list; B: IFS curves on LASSO feature list; C: IFS curves on LightGBM feature list; D: IFS curves on MCFS feature list; E: IFS curves on RF feature list; F: IFS curves on XGBoost feature list. Figure S2: IFS curves for evaluating the performance of two classification algorithms based on the weighted F1 score in cardiomyocytes. A: IFS curves on CatBoost feature list; B: IFS curves on LASSO feature list; C: IFS curves on LightGBM feature list; D: IFS curves on MCFS feature list; E: IFS curves on RF feature list; F: IFS curves on XGBoost feature list. Figure S3: IFS curves for evaluating the performance of two classification algorithms based on the weighted F1 score in endothelial cells. A: IFS curves on CatBoost feature list; B: IFS curves on LASSO feature list; C: IFS curves on LightGBM feature list; D: IFS curves on MCFS feature list; E: IFS curves on RF feature list; F: IFS curves on XGBoost feature list.
