# Supplementary figures and images for: Machine Learning in Identifying Marker Genes for Congenital Heart Diseases of Different Cardiac Cell Types
Source: Life (Basel). 2024 Aug 19;14(8):1032. doi: 10.3390/life14081032 (PMC11355424; doi:10.3390/life14081032)

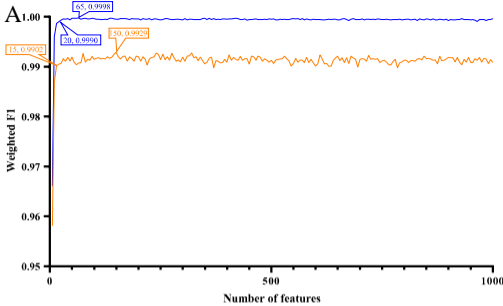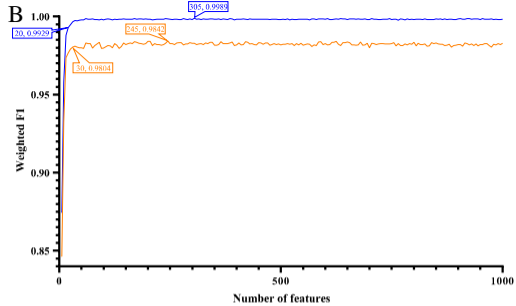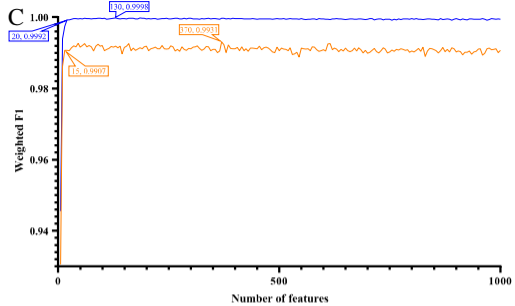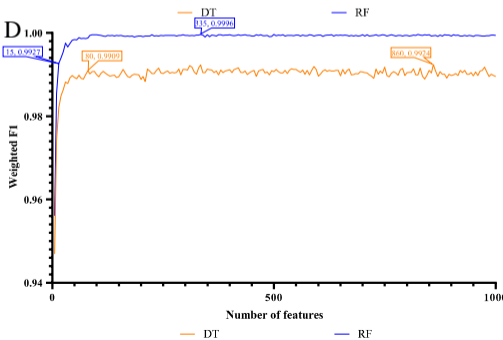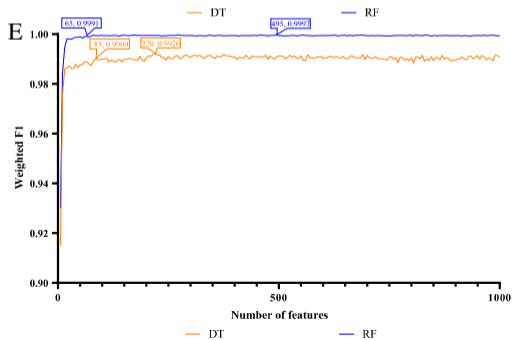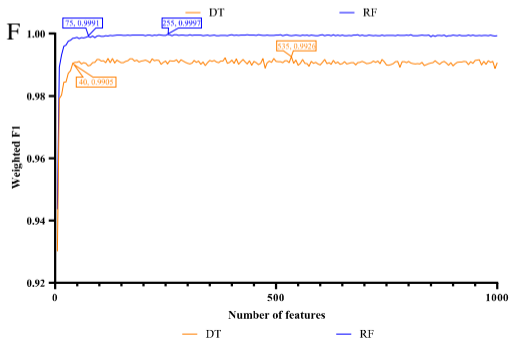

Supplement: Supplementary file 1 [file life-14-01032-s001.zip › Figure S1.pdf]

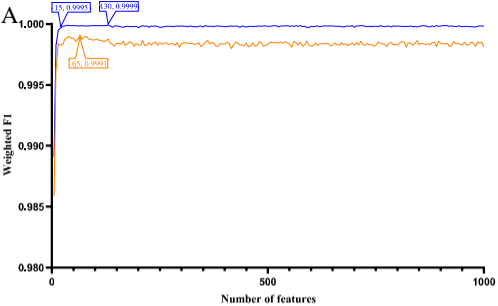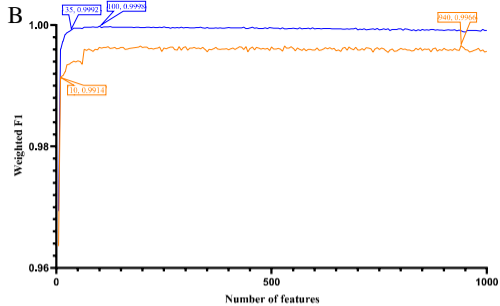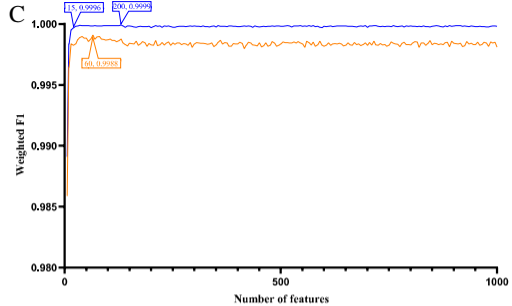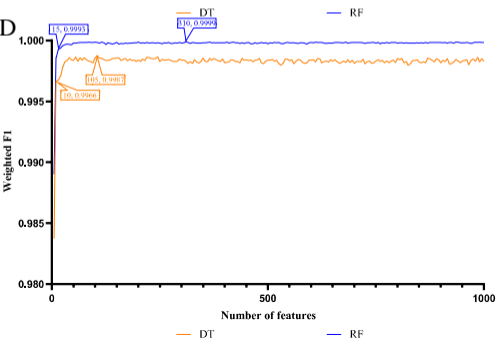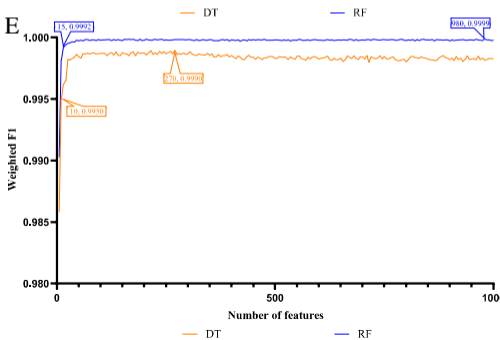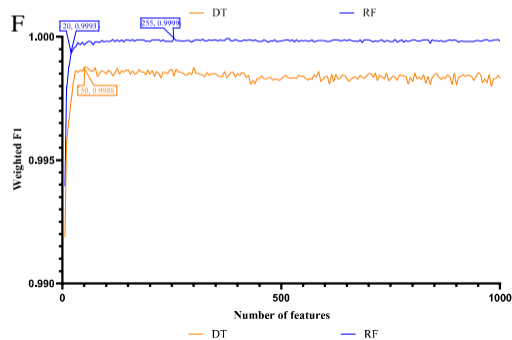

Supplement: Supplementary file 1 [file life-14-01032-s001.zip › Figure S2.pdf]

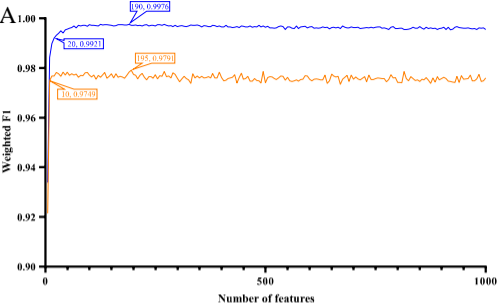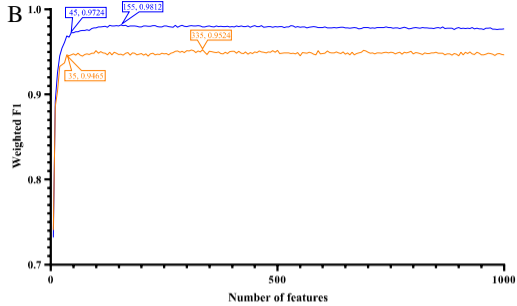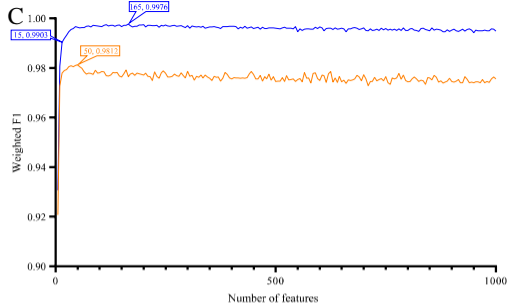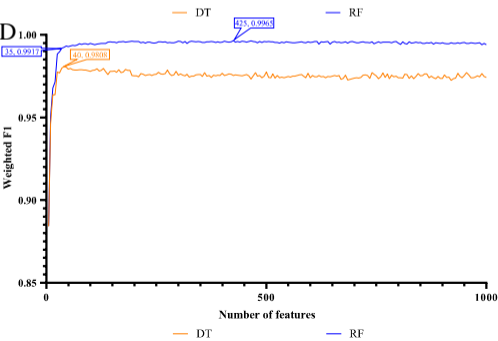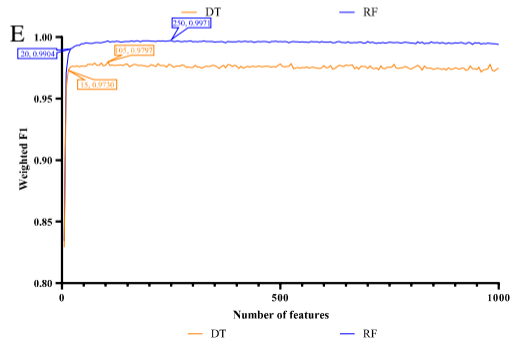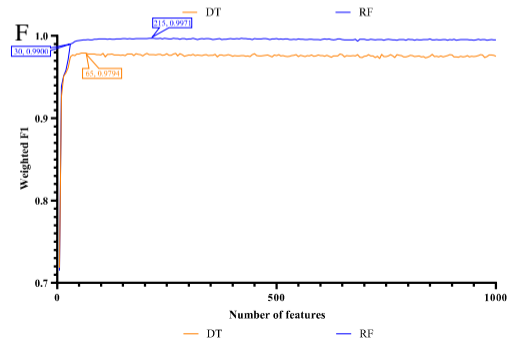

Supplement: Supplementary file 1 [file life-14-01032-s001.zip › Figure S3.pdf]
